# Ethnic minorities and prescription medication; concordance between self-reports and medical records

**DOI:** 10.1186/1472-6963-6-115

**Published:** 2006-09-13

**Authors:** Ellen Uiters, Liset van Dijk, Walter Devillé, Marleen Foets, Peter Spreeuwenberg, Peter P Groenewegen

**Affiliations:** 1 Netherlands Institute for Health Services Research (NIVEL), Utrecht, Netherlands; 2 Institute of Health Policy and Management, Erasmus Medical Centre, Rotterdam, Netherlands

## Abstract

**Background:**

Ethnic differences in health care utilisation are frequently reported in research. Little is known about the concordance between different methods of data collection among ethnic minorities. The aim of this study was to examine to which extent ethnic differences between self-reported data and data based on electronic medical records (EMR) from general practitioners (GPs) might be a validity issue or reflect a lower compliance among minority groups.

**Methods:**

A cross-sectional, national representative general practice study, using EMR data from 195 GPs. The study population consisted of Dutch, Turks, Surinamese, Antilleans and Morrocans. Self-reported data were collected through face-to-face interviews and could be linked to the EMR of GPs. The main outcome measures were the level of agreement between annual prescribing rate based on the EMRs of GPs and the self-reported receipt and use of prescriptions during the preceding 14 days.

**Results:**

The pattern of ethnic differences in receipt and use of prescription medication depended on whether self-reported data or EMR data were used. Ethnic differences based on self-reports were not consistently reflected in EMR data. The percentage of agreement above chance between EMR data and self-reported receipt was in general relative low.

**Conclusion:**

Ethnic differences between self-reported data and EMR data might not be fully perceived as a cross-cultural validity issue. At least for Moroccans and Turks, compliance with the prescribed medication by the GP is suggested not to be optimal.

## Background

The use of prescriptions is an important aspect of medical consumption in all western countries. Nevertheless, large differences in prescription use exist within countries. Ethnic minority groups are in general found to differ from the Dutch population in the use of prescription medication as well as over the counter medication (OTCs) [1-4]. These differences are often explained by cultural factors,(Table 1). A complicating factor in the comparison of figures concerning prescription utilisation, however, is the fact that they are often based on different methods of data collection. Data are typically obtained from patient interviews, self-reported surveys, pill counts, medical records or claims databases. Data obtained from these different sources often not correspond. In the literature, this finding is in general perceived as a validity issue. Consequently, validity of the results is examined by comparing one method of data collection with another method [5,6]. Regarding survey data collected among ethnic minorities this comparison can be complicated by the possibility of culturally determined information bias. Language problems, poorer recall of people and cultural differences (like the perception of time) are suggested to affect the validity of self-reported data from minority groups [7-9]. Furthermore, in health care research the design of questionnaires for use among ethnic minority groups often not address important aspects as salience of contents, equivalence of concepts and the use of comprehensible language for the less well educated [10]. Nevertheless, in addition to explaining disconcordance between several methods of data collection as a (cross-cultural) validity problem, the possibility that different methods of data collection may provide different outcomes received little attention. Self-reported use of prescription, for instance, might measure prescription use accurately but reflect something different than for instance prescription information retrieved from medical records. In other words, the fact that a patient received a prescription from the GP does not necessarily have to correspond with the self-reported use. A lower self-reported use could for instance be related to a lower compliance among minority groups than among the Dutch population.

In the Netherlands general practitioners (GPs) often assume that the expectation to receive a prescription after consultation is higher among ethnic minority groups than among the Dutch [11]. This picture is reflected in the higher prescription rates for ethnic minorities based on electronic medical records (EMR) than the rates of the Dutch population [12]. Research, however, showed that ethnic minorities often do not share this general perception of GPs and feel that they receive a prescription too easily. Moreover, dissatisfaction was expressed by the type of prescription prescribed; ethnic minority groups felt they received too often paracetamol [11]. These findings could negatively influence the compliance rate among the minority groups [13-15]. In our study we will explore to which extent ethnic differences in EMR data from GPs are concord with ethnic differences regarding self-reported prescription use. Focus will be on the question whether a possible disconcordance might be related to a different compliance among ethnic minorities. The level of agreement between EMR data and two self-reported measures of prescribed medication will be analysed. First, the agreement between EMR data and self-reported use of prescribed medication will be examined. Furthermore, the agreement between EMR data and self-reported receipt of a prescription will be analysed. If the level of agreement between EMR data and self- reported receipt of a prescription is higher than between EMR data and self-reported use, this would be an indication that ethnic minorities use less medication than is being prescribed. Consequently, this lower compliance would provide, in addition to the (cross-cultural) validity approach, an alternative explanation for disconcordance between ethnic differences in self-reports and EMR data concerning prescription use.

## Methods

### Data collection

Data collection was performed within the framework of the Second National Survey of General Practice. This survey was carried out in 2001 [16]. In our study, 195 GPs from 104 practices participated. The total patient population of these practices consisted of 385.461 people. Our study is based on a linkage of data from a survey and from the EMRs of GP practices. These data could be linked by means of a unique anonymous patient and practice identifier. In the participating general practices, 1.794,560 million contacts with patients during one year were recorded in the practice computer. In 57.4% of these contacts medication was prescribed [17]. The study was carried out according to Dutch legislation on privacy. The privacy regulation of the study was approved by the Dutch Data Protection Authority. According to Dutch legislation, obtaining informed consent is not obligatory for observational studies. Social -demographic characteristics of all listed patients were assessed by means of a census. This census also provided information about the country of birth of the patients and his or her parents. Information about the country of birth was used to indicate the ethnic background. When at least one parent was born abroad, a patient was indicated as having a foreign background [18]. The questions from the census were send in four languages (Dutch, Turks, Arabic (Moroccan) and English), accompanied by an inviting letter from their GP. Returning the census included informed consent. Data about self-reported receipt and use of prescriptions were collected through face-to-face interviews. People were interviewed at home. First a random sample per practice, totally 12.699 Dutch-speaking people, was interviewed, regardless of ethnic background. The response rate of this study was 64.5%. Response rate did not vary in an important way for age and sex. Refusal was the most common reason of non-response (66.9%).

An additional study was executed among a random sample of 1339 patients from the four largest minority groups in the Netherlands, originating from Turkey, Surinam, Morocco and the Netherlands Antilles. The content of the interviews among the minority groups was similar to the interviews among the Dutch-speaking respondents. To improve the validity and reliability of the questions, much attention was paid to the design of the questionnaire. The questionnaire was independently translated forward-backward for this purpose. As Surinam is a former Dutch colony that gained independence in 1975 and the Netherlands Antilles is still part of the Dutch Kingdom, people from these countries are familiar with the Dutch language. A pilot was performed to test comprehensibility and acceptance of the questionnaire on a comparable sample. Given the fact that bi-lingual people often are found to be biased by age, gender, education, producing translations that are too formal and literary for most people, field testing focussed on bi-linguals as well as mono-linguals [10,19]. The pilot interviews were observed on a screen by two members of the research team. This way questions needing clarification or causing any kind of an emotional response were notified and necessary adjustments could be applied. Interviewers were bilingual and received instruction training. The interviewers offered the opportunity to choose between an interview in Dutch or in the mother tongue of the respondents depending on the language mastery and preference. The response rate among the minority groups was 49.9%. Non-response rate was equal in the minority groups. Only women and elderly with a Surinam or Antillean background were relatively over represented in the study population. Difficulty to reach respondents (24.9%) and refusal (19.5%) were the major reasons of non-response.

### Measurements

EMR data about prescription medication were recorded by GPs. This procedure was part of the normal registration system of GPs. Based on these EMR data the percentage of patients who received a prescription during the past year was computed. This EMR information about prescribed medication was linked to the survey data. In the survey, people were asked whether they had used prescription medication. To reduce the possibility of recall bias, use of prescriptions was asked regarding the preceding 14 days. Because the use of OTC medication can possibly serve as a substitution for prescription medication, information about the use of OTC medication was also collected. Furthermore, people were asked whether a prescription had been prescribed to them during the preceding 14 days. People from the minority groups were also asked whether they retrieved OTC medication that cannot be bought without a prescription in the Netherlands while they visited their country of origin during the past year. People were asked not to take prescriptions received during a hospital admission and contraceptive prescriptions into account.

### Analyses

The analyses reported in this article are restricted to people aged at least 18. Respondents were only included if their survey date fell within the registration period of the GP. In total, 6363 people from the Dutch population, 189 Moroccans, 230 Turks, 89 Antilleans and 141 Surinamese satisfied these inclusion criteria. EMR data regarding contraceptives were excluded from the analyses. An indication of the extent in which ethnic differences in self-reported prescription use are concord with ethnic differences regarding EMR data was retrieved by computing the percentages of self-reported receipt and use of prescriptions and EMR data regarding prescriptions for each ethnic group (Table 2). Significant differences between the Dutch population and the minority groups were tested using logistic regression analyses (Table 2). The Dutch population served as the reference group. Subsequently, the level of agreement between self-reports and EMR data was examined more in detail by computing the percentage of agreement and disagreement between self-reports and EMR data (Table 3). Two aspects could be identified regarding agreement. Agreement was established in case the respondent reported to have received a prescription during the preceding 14 days and the EMR data showed that the GP actually had prescribed a prescription during the 14 days preceding the interview. Agreement was also established when according to the self-reports and EMR data no prescription had been prescribed. Disagreement was established in case the self-reports were not reflected in the EMR data. Respondents were classified as underreporting when based on self-reports no prescription was received, whereas the EMR data showed that the GP had prescribed medication. On the other hand, respondents were defined to overreport when the self-reported receipt of a prescription was not reflected in the EMR data. To account for the level of agreement to be expected by chance, (Cohen's) kappa was computed for each ethnic group.

## Results

Self-reported data concerning the receipt and use of prescriptions varied across the ethnic groups (Table 2). Compared to the Dutch population, people with a Surinam background were most likely to report the receipt of a prescription. People were also asked whether they had used prescription medication during the preceding 14 days. Again, Surinamese appeared to be most likely to have used prescription medication, although not significantly different from the Dutch population. Moroccans and Turks answered significantly least frequent to have used a prescription. With respect to the use of OTC medication, Antilleans, Surinamese and Dutch mentioned equally frequent to have used this type of medication. The minority groups were furthermore asked if they had bought medication that cannot be retrieved in the Netherlands without prescription during the last year in their country of origin. Moroccans and Turks confirmed this most frequently. Nevertheless, this only concerned a rather small number of people. None of the people with a Surinam background retrieved medication from their country of origin.

In addition to the self-reported information, data based on the GPs' EMR was analysed. According to these EMR data, medication was significantly most frequently prescribed to people with a Moroccan and Turkish background as compared to the Dutch population. These ethnic differences in EMR prescription rates did not completely concord with the ethnic differences based on self-reports. This was especially found among Moroccans. According to EMR data, Moroccans were most likely to have received a prescription, whereas self-reported use of medication was least frequently mentioned. Among the Dutch population the relatively high level of self-reported use of prescriptions did not concord with the relatively low level of EMR prescriptions as compared to the other groups.

The concordance between self-reports and EMR data was analysed more in detail by computing the level of agreement between the two methods of measurement (Table 3). With respect to the agreement between the self-reported receipt of a prescription and the EMR data, the highest level of agreement was found for the Dutch population. In 78.3% of the cases, self-reports corresponded with the EMR data. The lowest level of agreement was found for Surinamese (69.5%) and Antilleans (70.8%). Similarly, total disagreement was the highest in these latter two groups. The likelihood of underreporting the receipt of a prescription was the highest among the Dutch population and Antilleans. All minority groups appeared to be more likely to overreport the receipt of a prescription as compared to the Dutch population. Nevertheless, kappa varied from 0.10 among Moroccans to 0.26 among Turks. These relatively low kappa scores indicate that the agreement corrected for chance is generally low.

Similarly as computing the level of agreement between self-reported receipt of a prescription and EMR data, the level of agreement between self-reported use of a prescription and EMR data was examined. In contrast with the foregoing, the level of agreement was found to be the highest in the minority groups, except Surinamese. For Surinamese the level of agreement appeared to be lowest for both self-reported measures. This disagreement could mostly be attributed to the overreporting of receipt or use of a prescription within the Surinamese group. For all groups the level of agreement between self-reported prescription use and EMR data was lower than the level of agreement between self-reported reception of prescription and EMR data. However, taken into account the degree of agreement by chance, kappa scores were a little higher than the kappa scores between self-reported receipt of a prescription and EMR prescribed medication. Nevertheless, kappa scores were again relatively low, ranging from 0.16 among Moroccans to 0.23 among the Dutch population.

## Discussion

The results of this study show differences in prescription receipt and use among ethnic groups, regardless of the measure used. The pattern of these ethnic differences depended on whether self-reported data or EMR data were used. Ethnic differences based on self-reports were not consistently reflected in EMR data. Based on self-reported data, the minority groups were most likely to have received a prescription, whereas the self-reported use of prescriptions was relatively high in the Dutch and Surinamese population. The relatively high EMR prescription rate among minority groups was, especially for Turks and Moroccans, not reflected in a relatively high self-reported use of prescriptions. This suggests that although these two groups received relatively more prescriptions than the other groups from their GP, they seem to use least. OTC medication is not likely to substitute prescription medication, because among Moroccans and Turks this type of medication is less often used than in the other groups. The effect of medication retrieved from the country of origin is also presumably negligible, because this applies to only a rather small number of Moroccans and Turks.

The interpretation of the results is complicated by the fact that, taken into account the degree of agreement expected by chance, little agreement between EMR data and self-reported data was found. Conclusions concerning the adequacy of self-reported data about prescription medication in relation to EMR data cannot be drawn. The relatively low level of agreement after adjustment for chance could be attributed to actual differences between prescribing by GPs and the actual receipt and use of medication by patients but also to a low validity of self-reported data. However, the level of agreement did not differ systematically between the ethnic groups, implying that the validity of self- reported data concerning use and receipt of prescriptions does not differ among the ethnic groups. This suggests that the disconcordance between self-reports and EMR data among ethnic groups cannot be totally attributed to cross- cultural validity related explanations, like a cultural propensity to answer in a particular way. Given the comparable level of agreement between the ethnic groups, it might therefore be possible that the disconcordance between both methods of data collection reflect an actual difference in the receipt and use of prescriptions. In other words, compliance might be lower among the ethnic groups than the Dutch population. Little research in this field has yet been performed. One study among among men comparing self-reported use and registration data from a local insurance company over a 3 months period concluded in contrast to our findings that concordance was generally fair (kappa 0.60 among Dutch born and 0.54 among foreign-born) [20]. Future research will need to address this issue more in detail, unravelling possible mechanism explaining the level of agreement between self-reported data and EMR data.

Some disagreement between self-report data and EMR data is to be expected. Because immediate use is not always necessary after receiving a prescription, self-reported use of prescription medication and EMR data will not totally agree. Furthermore, some disagreement might be related to the fact that medication can be used without being prescribed recently. Therefore, even in case of a perfect self-report of use, concordance between EMR data and self-reports will not be perfect. Moreover, some limitations of our study might also negatively have influenced our comparison of EMR data and self-reports between ethnic groups. First, it was not possible to make a distinction in the self-reported data between medication prescribed by the GP or by a medical specialist. Repeat prescriptions from the medical specialist will usually be registered in the EMR from the GP, but not the first prescriptions received from the medical specialist. This could have resulted in some overestimating based on self-reports compared to the EMR data from the GP. Furthermore, registration in the EMR by the GPs might be incomplete, yielding a lower level of agreement. Nevertheless, analyses in a sub sample of practices satisfying important quality indicators for registration appeared not to result in systematically different findings.

## Conclusion

In conclusion, it remains unclear which underlying mechanism can explain the differences between the ethnic groups in EMR data and self-reported data. In general, above chance little agreement was found between EMR data and self-reported data. To enhance adequate prescribing and use of medication, future research should focus on explanations for these findings. The cross-cultural validity approach does not seem to be able to fully explain ethnic differences between self-report data and EMR data regarding prescription use. It could be that, at least for Moroccans and Turks, compliance with the prescribed medication by the GP is not optimal. It would be interesting to study to which extent the differences between ethnic groups are related to the level of acculturation. Does for instance the level of agreement improves as the level of acculturation increases? Nevertheless, evidence for a lower compliance among minority groups requires more attention for compliance enhancing methods and for the efficiency of the prescribing patterns of GPs. Consults that ended without mutual agreement more often resulted in non-compliance with prescribed therapy among patients with an ethnic-minority background [21]. Mutual agreement requires a clarification of the patients expectations concerning prescriptions. The finding that ethnic minorities in the Netherlands felt that they received a prescription too easily and were dissatisfied with the type of medication prescribed is an indication that this mutual understanding between GP and patients with a minority background can be improved.

## Competing interests

The author(s) declare that they have no competing interests.

## Authors' contributions

Obtained research funding: MF and PG

Coordination data collection: MF, PG, WD

Statistical analyses: EU, LvD and PS

Manuscript draft: EU, LvD, MF, WD, PG

All authors read and approved the final manuscript.

## Pre-publication history

The pre-publication history for this paper can be accessed here:



## Figures and Tables

**Table 1:** Consumption of medication in the Netherlands

Dutch GPs are known for being reluctant to prescribe compared to doctors in other European countries [22]. Many patients share this reluctant attitude. They are rather relieved when medication turns out to be unnecessary after a consultation with their doctor. Compared to other European countries the use of medication in the Netherlands is low and OECD-data showed that the Netherlands has one of the lowest expenditures on prescribed medication [22]. Nevertheless; the use of medication in the Netherlands has increased substantially over the last decade. In 2001, the year of the second National Survey of General Practice, expenditures on medication sold in public pharmacies, increased by 11% [23].

**Table 2:** EMR data and self-reports concerning prescriptions across the ethnic groups*

	**Dutch**	**Morocco**	**Turkey**	**Netherlands Antilles**	**Surinam**
**N**	6363	189	230	89	141
**self- reported data**					
receipt of prescribed medication during the past 14 days (%)	15.1	**23.8**	**29.4**	**24.7**	**31.9**
use of prescribed medication during the past 14 days (%)	48.2	**38.1**	**39.1**	41.6	53.9
use of OTC medication during the past 14 days (%)	37.4	**30.3**	**26.1**	32.6	34.3
retrieved medication in country of origin (%)	not applicable	3.8	3.4	1.1	
**EMR data**					
prescribed medication during the past year (%)	76.1	**85.2**	**84.3**	77.5	81.6

* significant difference with the Dutch population are printed in bold (p < 0.05)

**Table 3:** Comparisons among self-reports and EMR data by ethnic group

	**Dutch**	**Morocco**	**Turkey**	**Netherlands Antilles**	**Surinam**
**self-reported receipt of a prescription vs. EMR prescribed medication**					
Percentage agreement					
both yes	4.5	3.7	10.1	5.6	9.9
both no	73.8	70.4	62.7	65.2	59.6
Total agreement	78.3	74.1	72.8	70.8	69.5
Percentage disagreement					
underreporting	11.2	5.8	7.9	10.1	8.5
overreporting	10.6	20.1	19.3	19.1	22.0
total disagreement	21.8	25.9	27.2	29.2	30.5
kappa	0.16	0.10	0.26	0.11	0.21
**self-reported use of prescribed medication vs. EMR prescribed medication**					
Percentage agreement					
both yes	13.2	6.9	11.7	11.2	15.6
both no	49.3	59.3	54.8	53.9	43.3
total agreement	62.5	66.2	66.5	65.1	58.9
Percentage disagreement					
underreporting	2.5	2.6	6.1	4.5	2.8
overreporting	35.0	31.2	27.4	30.3	38.3
total disagreement	37.5	33.8	33.5	34.8	41.1
Kappa	0.23	0.16	0.22	0.21	0.22
